# Data Note: The Digital Ludeme Project Database

**DOI:** 10.12688/openreseurope.16524.1

**Published:** 2023-09-28

**Authors:** Cameron Browne, Matthew Stephenson, Walter Crist

**Affiliations:** 1 Maastricht University, Maastricht, Limburg, The Netherlands

**Keywords:** Games Studies, Ancient Board Games, Ludemes, Digital Humanities, Cultural Computing, FAIR Data Principles

## Abstract

This document outlines the types of data collected for the Digital Ludeme Project, an ERC-funded research project that aims to improve our understanding of the development of games throughout human history through computational analysis of the available (partial) historical data of games. This document outlines how this data is collected, formatted and stored, and how it can be accessed. It is the aim of the Digital Ludeme Project to provide a data resource of unprecedented depth and scope for the benefit of historical games researchers worldwide. Special attention is paid to the FAIR Guiding Principles for scientific data management and stewardship.

## Introduction

The Digital Ludeme Project is a five-year research project involving the analysis and reconstruction of ancient and historical games from incomplete descriptions, artistic depictions, and archaeological evidence using modern computational techniques ^1^. The project focuses on games that rewards mental acumen over physical skill. Generally, these broadly fit what are commonly known as board games, card games, and domino games. The goal of the project is to document 1,000 of these games which were important for the historical development of games, across the world, and throughout history. Some outliers exist, e.g. chance-based games such as Gyan Chaupar and the Game of the Goose that are still obviously culturally important.

To achieve this, a digital database of evidence for ancient and traditional games is required, drawing from multiple disciplines and in various formats; this is the first time that such a collection of evidence has been attempted for such a wide range of games and from such a wide range of perspectives and sources. This document describes how we are obtaining this data and storing it in a consistent format both for use within the Digital Ludeme Project and for the benefit of other researchers and practitioners, and how we are adopting the FAIR principles to make this database as reliable, accessible and useful as possible.

This document is an updated version of the “Digital Ludeme Project Database Guide” ^2^ that reflects changes made to the current version of the database 1.3.12. ^1^ Most importantly, these include the addition of a “ludemes” table that provides information about which ludemes — i.e. units of game-related information – are available in which games and rulesets in the database.

### Purpose

To study traditional games, it is necessary for a rigorous approach to compile and analyse what is known about them. Historically, games research has often relied on works such as Murray’s History of Board Games other than Chess ^3^, Bell’s Board and Table Games from Many Civilizations ^4^ and Parlett’s Oxford History of Board Games ^5^. These works are largely compilations of primary research performed by anthropologists, historians, and people interested in games, but which often include assumptions, and, not infrequently, mistranslations and incorrect descriptions of the original sources. Other authors provide reconstructions for games based on their assumptions about how games may be similar to one another. To approach the reconstruction of games empirically, the Digital Ludeme Project uses primary sources to identify exactly what is known about the relevant games, in order to provide accurate reconstructions that is as reflective as possible of the current knowledge about these games. The database resulting from this research not only serves to facilitate game reconstruction, but is the most rigorous and complete representation of games – as well as being the most honest in terms of what we actually know about games and not what is assumed to the true – available to researchers and others with an interest in games, and has the potential to become the most comprehensive traditional games database in the world. In the context of this paper, we refer to this collection of data generated by the Digital Ludeme Project as the “DLP Database”.

## Methods

### Conceptual Structure

**Figure 1.**
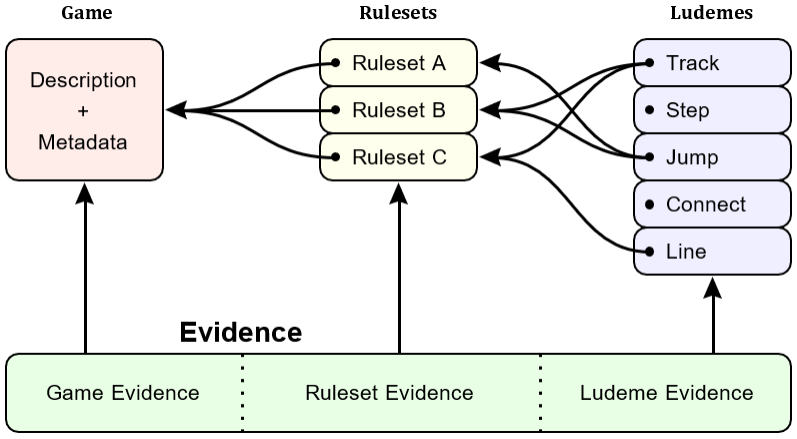
Overview of the main data groups and the relationships between them.

In order to operationalise this research framework in a database that captures the inherent variations that exist in the ways games are played, and to take into account both functional and cultural definitions of games, the DLP Database conceptualises games in an innovative way, that allows for the identification and corroboration of evidence with specific ways that games are played. As a result, the data comprising the DLP Database can be decomposed into two main types: game-related and evidence-related. These conceptual definitions are broadly defined and will necessarily inform each other, as shown in Figure 1; each game is composed of a number of rulesets, each of which is defined by component ludemes, and there exist separate types of evidence for each of these conceptual groupings. These two main types of data can be further decomposed into the following groups.

***Ludemes.*** A ludeme is a single elemental building block of a game. Multiple ludemes can be combined together to create a description of a specific piece of equipment or rule that a game uses ^6^. Because of this each Ruleset, and by extension every game, within the DLP database can be described using these ludemes in a single plain text file called a ludeme description (with the extension “.lud”). Each ludeme is stored in the DLP database and is associated with the Rulesets which use it. If a game has any ruleset that uses a particular ludeme, then by extension, that game will also be considered as using that ludeme.

### Game-Related

The games-related data refers to individual instances of games, their rulesets, and the ludemes that make them up. Much of this type of data is derived from the evidence-related categories – although not exclusively – and is indirect in nature.

***Games.*** Games can broadly be described as being played by rules which are distinct from other games, but which also are strongly tied to cultural histories and practices. Therefore, a ruleset alone is not sufficient to define a game, though a game must consist of at least one ruleset. A single game can often have multiple distinct rulesets. Cultural information drawn from primary sources helps to determine when a single game should have multiple rulesets or whether these should be separate games.

Each game entry in the database collects the known rulesets for that game in a single access point. The decision between whether two variants of a given game should be defined as different games or as variant rulesets of the same game is guided by the historical and cultural context of those variants and is not always clear cut. For example, the game Alquerque contains two rulesets, Alfonso X and Covarrubias, because they both describe the game as played in Spain, but with some rule differences. However, we distinguish between the Greek game Portes and English Backgammon as two distinct games despite their nearly identical rules, due to significant geographic and cultural differences.

Games can have multiple names. We use the name for the game as used by the culture where it is most popular, where that can be determined, and list alternative known games in the aliases field.

This data group constitutes our high-level description of games.

***Rulesets.*** Rulesets are the set of ludemes that describe the specific equipment and rules associated with them. Rulesets can be functionally defined, where changes such as a larger board or a different scoring system would typically not be sufficient for being described as a different ruleset. In the case of Alquerque, the two rulesets differ in that the Covarrubias ruleset imposes a mandatory captured rule as well as the huff rule on top of those presented in the Alfonso X ruleset. However, separate rulesets may have nearly identical sets of ludemes, but must be defined separately because of the cultural information related to them. Backgammon and Portes, as discussed above, demonstrate nearly identical rulesets that are distinct because of their cultural context. There are different types of rulesets.

Games can have multiple alternative rulesets, and often these rulesets come from different kinds of sources. Some are described—explained by someone who belongs to the culture which plays the game, or someone who can be considered to be knowledgeable about the rules of the game, while others are observed—described by someone who recently learned or observed the game as played by others. Rulesets may also be suggested, where someone has provided rules for a game for which the original rules are lost; scholarly, where an expert on a particular traditional game has provided an informed set of rules for playing that game; or reconstructed, which will be rulesets compiled in the process of game reconstruction over the course of the DLP. Finally, incomplete rulesets are those which belong to games for which no complete rules are known to have survived, and are supplied from DLP evidence. Each known ruleset for a game is defined in terms of the ludemes that make it up, as well as the cultural information about who, when, and where it was played. Rulesets which are observed or described will typically be linked to evidence.

This data group constitutes our mid-level description of games.

This data group constitutes our low-level description of games.

### Evidence-Related Data

The evidence-related data refers to items of evidence from various sources that support the existence of given games, rulesets and ludemes. This type of data is direct in nature and typically comes from well defined sources.

***Defining Evidence.*** Because the scope of the project covers prehistory to the present day, there are many different relevant resources which provide information about games, rulesets, and ludemes. The relevant information is not restricted to the mechanics of how the games are played, but also includes geographic, chronological, and social data, which are essential for being able to model the spread of games. In many cases, a single piece of evidence may provide a complete picture of the ludemes and rulesets that make up a game as well as the relevant cultural material. In many other cases, the evidence may only describe a part of a game, and multiple pieces of evidence must be used for a single game to obtain a more complete picture. For games without complete known rulesets, such as Senet, as much of the evidence will be gathered as is possible. This will allow the project to evaluate what information is most strongly supported by evidence, and what portions of potential rulesets will require reconstruction. For other games which are well-understood and will not need reconstruction, such as Chess, collection of evidence cannot be comprehensive, but will be sampled to reflect the chronological, social, and geographic spectrum of that game, in order to aid in the reconstruction of other games and to accurately model the spread of that game and its potential relationship to other games.

***Types of Evidence.*** A piece of evidence is a “primary source of information” which provides some form of documentation situating a game in a particular time and/or place. A primary source of information provides first-hand information about a game. As such, the historical data collected on each piece of evidence are those which provide information about where, when, how, and by whom a game was played, not necessarily data about the document from which the information comes.

For example, when documenting relevant artefacts discovered during archaeological excavations, it is the place where they were found, and the dates ascribed to the artefacts, not the date of the archaeological project or the book or article they were published in, that is important. When recording data from evidence in a text published posthumously, the date the text was written is the important piece of information, not when it was published. For a text published somewhere other than the place it describes (such as most ethnographies), it is the place being described, not the place of publication or residence of the author that is to be recorded. In some cases, the data from a document and the game will be the same, such as in original texts discussing games as they were played at the time the text was written, as in the 13th century Libro de los Juegos of Alfonso X of Castille, which describes games played at that king’s court, as written by him. An exhaustive list of scenarios could fill volumes; what is important is to consider each piece of information that is entered, and to be certain that, as much as is possible, it reflects the information as it relates to the game as played in its relevant place and time.

Most pieces of evidence will also provide at least some information about the ludemes that make up the ruleset(s) of a game. Each kind of evidence has inherent strengths and weaknesses, and has the potential to provide different pieces of information about a game, ruleset, or ludeme.

The following categories cover the types of primary sources of information that qualify as evidence used for the DLP, though others may be added at a later date:

1. A rules text is a text which was solely written to explain the rules of a game as it was played during the time in which it was written. A good example of this would be the description of Alquerque in Alfonso X’s Libro de los Juegos.
2. A contemporary rule description is a text that contains the rules of a game, though the subject of the text as a whole may focus on another topic, by someone from the culture to which a game belongs and who lived during the time the game was played.
3. A historical rule description is a text which purports to tell the rules about a game in the past, from the perspective of the text’s author. An example of this would be a text describing how the rules of a game were different a century before the author’s time.
4. A contemporary text is a text that contains some information about a game, written during the time the game was played, but which does not attempt to describe the rules of the game. An example of this would be a text that mentions the favourite game of a king, or a law that forbids a certain game.
5. A historical text is a text that contains some information about a game that was played in the past, with respect to the author, but which does not attempt to describe the rules of a game. An example of this would be the passage in the Persian epic poem Shahnameh, which features a story about the kings of Persia and India playing Nard and Shatranj against each other.
6. An ethnography is a description or mention of a game by a person talking about a culture to which they do not belong. Though this account is written, it is not a text because it is the content of the encounter which constitutes the evidence. This may be an anthropologist’s account of game playing among a group of people they are studying, rules reported by someone who was taught a game by a person from another country, or anyone describing a game from another culture than their own.
7. An artefact is an object that was used to play a game, such as a board, a set of pieces, a pack of cards, or dice. These can tell us about the equipment that was used to play a game, and when they come from a well-documented archaeological context, other information such as date, location, and information in social categories may also be inferred. An example of this are the Senet game paraphernalia found in the tomb of Tutankhamun.
8. An artistic depiction is a representation of a game or people playing a game in a visual media format. Sculptures or paintings of people playing games, where the game can be identified, would be examples of this.

Other kinds of information which come from secondary sources – those which discuss and analyse information from primary sources – , including many books, articles, pamphlets, or websites, are not themselves evidence, but may be cited in an entry for a piece of evidence when they provide relevant information regarding the culture, time, or place about when a game was played.

***Criteria for Inclusion.*** Evidence is only collected for games which are included in the DLP. Because the DLP seeks to model the spread of traditional games, it is important to be able to track what is known about these games in a given place and time. Traditional games are primarily learned from another person, rather than through rules codified in a document. There is, therefore, some grey area in the determination that must be made for inclusion of a game in the DLP. While this social definition is the conceptual key to modelling the spread of games, it is not always immediately clear when to determine that a game should be included based on the kinds of evidence that are available to us. We therefore use the following criteria when determining when to include evidence for a game in the database:

1. The game can be documented before the nineteenth century.
  – The Industrial Revolution, and the commercialisation that followed, made vast changes in the ways games were created and learned, and thus most games invented after this point are not considered eligible. For example, Halma, Chinese Checkers, and Monopoly are not included because they were games that were invented at a clear point in time and marketed commercially, thus spreading through different mechanisms than those modelled in this project. But, games which can be shown to have existed before this process can be inferred to have spread and become popular through the kinds of social mechanisms relevant to this project.
  Nevertheless, there are many relevant games which have only been documented since the nineteenth century
  – though they may be much older –, which are important for analysis. To include such games, the following criteria should be taken into consideration as proxies for determining a game’s inclusion.
2. A game must be non-proprietary.
  – For example, Monopoly, though widely played and often learned from other players, is disqualified because its owner has control over where it is sold, what the rules are, and other factors that make it unfit for our research.
3. A game only documented after 1800 cannot have an identified inventor.
  – Though every game must have been invented by someone, most games that were invented during and since the nineteenth century have spread due to the commercial sale and marketing of games. When a game predates those processes, it can be inferred that it spread through the types of social contacts modelled in this project. Many games predating the nineteenth century have purported and some may even have documented inventors, but the method by which they spread is different.
4. It must be reasonably demonstrable that a game has some cultural value within the society in which it is played.
  – This is purposefully widely defined, and there are a multitude of ways this can be shown. The absence of a commercial game set of that game is one clue, but also statements which indicate that the game has a history in that culture, that it is generally learned from other people, the fact that similar games have been documented in neighbouring regions, or that when asked to play a game people know it and do not need to be taught it, are ways to approach this. For example, the game Jekab, observed in the Marshall Islands in 2017 is played as part of cultural festivals on the islands, and is a part of daily life as it is commonly played in public places ^7^.

## DLP Data Format

**Figure 2.**
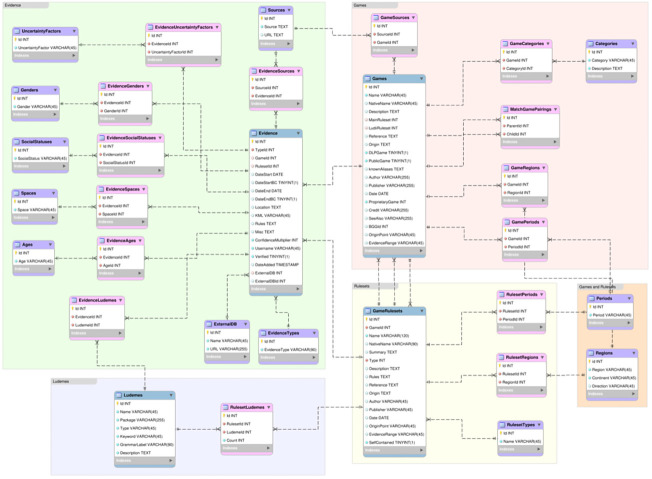
An Entity Relationship Diagram (ERD) of the DLP Database.

The DLP database is a MySQL-based database structure. Figure 2 shows an Entity Relationship Diagram (ERD) of the DLP database. Blue coloured tables signify Fact tables, purple coloured tables signify Dimension tables, and pink coloured tables signify Junction tables. The tables within the database can be conceptually split into five groups, Games, Rulesets, Games and Rulesets, Ludemes, and Evidence.

### Games Section

**Figure 3.**
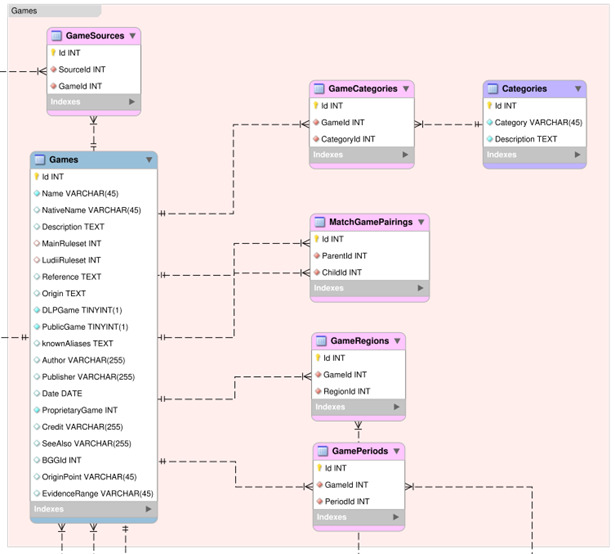
An Entity Relationship Diagram (ERD) for the Games group of the DLP database.

This section describes all the tables within the Games group of the DLP database, as shown in Figure 3.

***Games.*** Fact table, describing each game, as shown in Table 1.

- Id: Primary identifier (should be left blank).
- Name: Name of the game using only Ascii characters. This is the name of the .lud game description file associated with this game. The name should be a name for the game that is used by the culture in which it was most widely played, where this can be determined. It could be the most commonly- used name for a game, the most widespread name for a game, or the earliest name for a game, but it must in some way be connected to the culture from which it originates. Where the name of the game contains accents or other characters not used in English, an approximation should be used, and the correct name used in NativeName (see below).
- NativeName: Name of the game using any utf-8 valid characters. This is the name of the game that will be displayed on the ludii.games website. Leave blank if the native name for the game is the same as the name of its Ascii name. This is used for games with accents or other non-English characters.
- Description: This is a brief description about what is known about the game. Typically, it briefly describes where the game is played, what type of game it is, and any other interesting, relevant, or unique cultural information about the game that is known.
- MainRuleset: Primary identifier of the Ruleset table value associated with the base ruleset of this game piece. The base ruleset of this game is what will be displayed by default when viewing the game on the ludii.games website.
- LudiiRuleset: Primary identifier of the Ruleset table value associated with the default Ludii ruleset of this game piece. The default Ludii ruleset of this game is what will be loaded by default if the game is opened within the Ludii application viewing the game on the ludii.games website. This must be a playable ruleset.
- Reference: This field contains a reference for a game. Typically, this field contains works that discuss games, but which do not provide new evidence for the game. Murray’s History of Board Games other than Chess ^3^ is an example of a work that discusses many games, but for which it is not the primary source, and therefore should be included here.
- Origin: The origin is an estimation of the earliest place that the game was played, based on the evidence collected. It can be as specifically defined or as broadly defined as the evidence supports.
- DLPGame: True if this game is part of the Digital Ludeme Project.
- PublicGame: True if this game should be publicly available.
- knownAliases: Comma separated list of other known names for this game aside from the main Name chosen above.
- Author: Original author or designer of the game, where known.
- Publisher: Original publisher of the game, where known.
- Date: Original date that the game was released, for those where this is appropriate. Dates where the day or month is 00, means that the value for this is unknown.
- ProprietaryGame: True if this game is proprietary.
- Credit: The creator of the .lud game description file associated with this game.
- SeeAlso: This field references other games that are culturally, historically, or otherwise relevant to this game, and will display them on ludii.games.
- OriginPoint: GPS coordinates for the earliest piece of evidence existing in the database for this game, in the form (6◦44’29.95”N, 44◦15’43.61”E). This value is automatically updated by an external script and should usually be left blank. If you wish to override this script and set the origin point for yourself, then a ‘*’ must be added to the end of the OriginPoint value (e.g. 6◦44’29.95”N, 44◦15’43.61”E*).
- EvidenceRange: Time span of all evidence existing in the database for this game, in the form (EarliestYear, LatestYear). EarliestYear and LatestYear are given as the number of years after 3500BCE (i.e. 1500 BCE would be 2000, and 600 CE would be 4100). This value is automatically updated by an external script and should always be left blank.

**Table 1.** Structure of the Games table.

Field	Type	Null	Key	Default	Extra	Foreign Key
Id	int(11)	NO	PRI	NULL	auto increment	
Name	varchar(45)	NO	UNI	NULL		
NativeName	varchar(45)	YES		NULL		
Description	text	YES		NULL		
MainRuleset	int(11)	YES	MUL	NULL		GameRulesets – Id
LudiiRuleset	int(11)	YES	MUL	NULL		GameRulesets – Id
Reference	text	YES		NULL		
Origin	text	YES		NULL		
DLPGame	tinyint(1)	NO		1		
PublicGame	tinyint(1)	NO		1		
knownAliases	text	YES		NULL		
Author	varchar(255)	YES		NULL		
Publisher	varchar(255)	YES		NULL		
Date	date	YES		NULL		
ProprietaryGame	int(11)	NO		0		
Credit	text	YES		NULL		
SeeAlso	varchar(255)	YES		NULL		
BGGId	int(11)	YES		NULL		
OriginPoint	varchar(45)	YES		NULL		
EvidenceRange	varchar(45)	YES		NULL		

***GameSources.*** Junction table, describing pairings between games and sources (see Table 2). This ties a Source (i.e., a bibliographic reference) to a game.

- Id: Primary identifier (should be left blank).
- SourceId: Primary identifier of the Sources table value associated with this pairing.
- GameId: Primary identifier of the Game table value associated with this pairing.

**Table 2.** Structure of the GameSources table.

Field	Type	Null	Key	Default	Extra	Foreign Key
Id	int(11)	NO	PRI	NULL	auto increment	
SourceId	int(11)	NO	MUL	NULL		Sources – Id
GameId	int(11)	NO	MUL	NULL		Games – Id

***GameCategories.*** Junction table, describing pairings between games and categories (see Table 3). This ties a category (e.g. War, Hunt, Space, Card, Dice, etc.) to a game. These categories are based on similar clusters of core functionality between games.

- Id: Primary identifier (should be left blank).
- GameId: Primary identifier of the Game table value associated with this pairing.
- CategoryId: Primary identifier of the Categories table value associated with this pairing.

**Table 3.** Structure of the GameCategories table.

Field	Type	Null	Key	Default	Extra	Foreign Key
Id	int(11)	NO	PRI	NULL	auto increment	
GameId	int(11)	NO	MUL	NULL		Games – Id
CategoryId	int(11)	NO	MUL	NULL		Categories – Id

***MatchGamePairings.*** Junction table, describing pairings between different games based on if one game contains another as part of its own rules (i.e. the game of Tavli (parent) consists of players playing many repeated rounds of the game Portes, Fevga and Plakoto (children)). This is shown in Table 4.

- Id: Primary identifier (should be left blank).
- ParentId: Primary identifier of the Game table value associated with the parent game in this pairing.
- ChildId: Primary identifier of the Game table value associated with the parent game in this pairing.

**Table 4.** Structure of the MatchGamePairings table.

Field	Type	Null	Key	Default	Extra	Foreign Key
Id	int(11)	NO	PRI	NULL	auto increment	
ParentId	int(11)	NO	MUL	NULL		Games – Id
ChildId	int(11)	NO	MUL	NULL		Games – Id

***GamePeriods.*** Junction table, describing pairings between games and periods (see Table 5). This ties a period (e.g. Ancient, Medieval, Modern) to a game.

- Id: Primary identifier (should be left blank).
- GameId: Primary identifier of the Game table value associated with this pairing.
- PeriodId: Primary identifier of the Period table value associated with this pairing.

**Table 5.** Structure of the GamePeriods table.

Field	Type	Null	Key	Default	Extra	Foreign Key
Id	int(11)	NO	PRI	NULL	auto increment	
GameId	int(11)	NO	MUL	NULL		Games – Id
PeriodId	int(11)	NO	MUL	NULL		Periods – Id

***GameRegions.*** Junction table, describing pairings between games and regions (see Table 6). This ties a region (e.g. Northern Africa, Caribbean, Western Asia, etc.) to a game.

**Table 6.** Structure of the GameRegions table.

Field	Type	Null	Key	Default	Extra	Foreign Key
Id	int(11)	NO	PRI	NULL	auto increment	
GameId	int(11)	NO	MUL	NULL		Games – Id
RegionId	int(11)	NO	MUL	NULL		Regions – Id

- Id: Primary identifier (should be left blank).
- GameId: Primary identifier of the Game table value associated with this pairing.
- RegionId: Primary identifier of the Region table value associated with this pairing.

***Categories.*** Dimension table, describing possible categories for games (see Table 7). Games are categorised into groups based on the mechanics of play.

**Table 7.** Structure of the Categories table.

Field	Type	Null	Key	Default	Extra	Foreign Key
Id	int(11)	NO	PRI	NULL	auto increment	
Category	varchar(45)	NO		NULL		
Description	text	NO		NULL

- Id: Primary identifier (should be left blank).
- Category: Name of game category (e.g. War, Hunt, Space, Card, Dice, etc.).
- Description: Short description of the category.

### Rulesets Section

**Figure 4.**
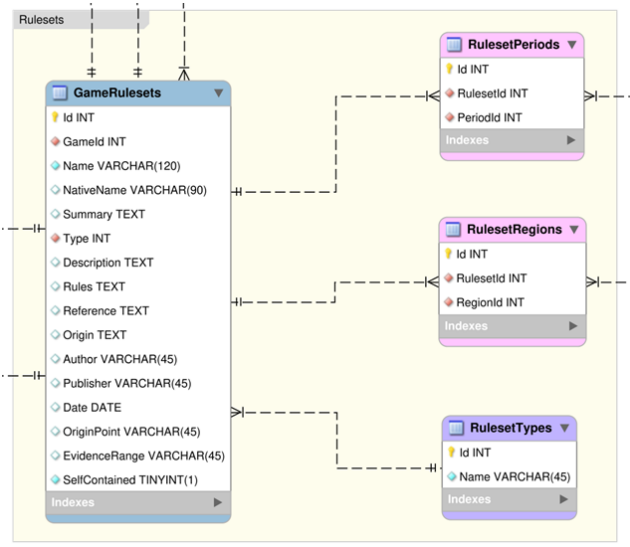
An Entity Relationship Diagram (ERD) for the Rulesets group of the DLP database.

This section describes all the tables within the Rulesets group of the DLP database, see Figure 4.

***GameRulesets.*** Fact table, describing each Ruleset (see Table 8).

**Table 8.** Structure of the GameRulesets table.

Field	Type	Null	Key	Default	Extra	Foreign Key
Id	int(11)	NO	PRI	NULL	auto increment	
GameId	int(11)	NO	MUL	NULL		Games – Id
Name	varchar(45)	NO		Default		
NativeName	varchar(45)	YES		NULL		
Summary	text	YES		NULL		
Type	int(11)	NO	MUL	1		EvidenceTypes – Id
Description	text	YES		NULL		
Rules	text	YES		NULL		
Reference	text	YES		NULL		
Origin	text	YES		NULL		
Author	varchar(45)	YES		NULL		
Publisher	varchar(45)	YES		NULL		
Date	date	YES		NULL		
OriginPoint	varchar(45)	YES		NULL		
EvidenceRange	varchar(45)	YES		NULL		
SelfContained	tintint(1)	YES		NULL

- Id: Primary identifier (should be left blank).
- GameId: Primary identifier of the Games table value associated with this Ruleset.
- Name: Name of the ruleset using only Ascii characters. This is the name that will be used within the .lud game description file associated with this ruleset. Where possible, this will be the name of this set of rules as they are called by the culture that plays them. In many cases, this will not be identified, so it can be given any name that distinguishes it from the other rulesets for this game.
- NativeName: Name of the ruleset using any utf-8 valid characters. This is the name of the ruleset that will be displayed on the ludii.games website. Leave blank if the native name for the ruleset is the same as its Ascii name.
- Summary: This is a brief summary describing what is unique about this ruleset to appear on the game’s page on ludii.games.
- Type: Primary identifier of the RulesetTypes table value associated with this ruleset. This assigns a Ruleset Type to a particular ruleset.
- Description: The ruleset description includes a brief description about the ruleset, and can include cultural information or information about who reported this ruleset.
- Ruleset: These are the rules of this ruleset, described with reference to the main ruleset for this game; i.e., only the differences between this ruleset and the main ruleset of the game should be described.
- Reference: Reference for the ruleset.
- Origin: The origin is an estimation of the earliest place that the ruleset was played, based on the evidence collected. It can be as specifically defined or as broadly defined as the evidence supports.
- Author: Original author or designer of the ruleset, where applicable.
- Publisher: Original publisher of the ruleset, where applicable.
- Date: Original date that the ruleset was released, where applicable. Dates where the day or month is 00, means that the value for this is unknown.
- OriginPoint: GPS coordinates for the earliest piece of evidence in the database for this ruleset, in the form (6◦44’29.95”N, 44◦15’43.61”E). This value is automatically updated by an external script and should usually be left blank. If you wish to override this script and set the origin point for yourself, then a ’*’ must be added to the end of the OriginPoint value (e.g. 6◦44’29.95”N, 44◦15’43.61”E*).
- EvidenceRange: Time span of all evidence in the database for this ruleset, in the form (EarliestYear, LatestYear). EarliestYear and LatestYear are given as the number of years after 3500 BCE (i.e. 1500 BCE would be 2000, and 600 CE would be 4100). This value is automatically updated by an external script and should always be left blank.
- SelfContained: True if the rules described for this ruleset do not require reference to the original parent game.

***RulesetPeriods.*** Junction table, describing pairings between rulesets and periods (see Table 9). This ties a period (e.g. Ancient, Medieval, Modern) to a ruleset.

**Table 9.** Structure of the RulesetPeriods table.

Field	Type	Null	Key	Default	Extra	Foreign Key
Id	int(11)	NO	PRI	NULL	auto increment	
RulesetId	int(11)	NO	MUL	NULL		GameRulesets – Id
PeriodId	int(11)	NO	MUL	NULL		Periods – Id

- Id: Primary identifier (should be left blank).
- RulesetId: Primary identifier of the Ruleset table value associated with this pairing.
- PeriodId: Primary identifier of the Period table value associated with this pairing.

***RulesetRegions.*** Junction table, describing pairings between rulesets and regions, as per Table 10. This ties a region (e.g. Northern Africa, Caribbean, Western Asia, etc.) to a ruleset.

**Table 10.** Structure of the RulesetRegions table.

Field	Type	Null	Key	Default	Extra	Foreign Key
Id	int(11)	NO	PRI	NULL	auto increment	
RulesetId	int(11)	NO	MUL	NULL		GameRulesets – Id
RegionId	int(11)	NO	MUL	NULL		Regions – Id

- Id: Primary identifier (should be left blank).
- RulesetId: Primary identifier of the Ruleset table value associated with this pairing.
- RegiondId: Primary identifier of the Region table value associated with this pairing.

***RulesetTypes.*** Dimension table, describing possible types for rulesets, as per Table 11.

- Id: Primary identifier (should be left blank).
- Name: Name of ruleset type. The ruleset types are as follows:
  – Described: A ruleset described by someone from the culture to which the game belongs.
  – Observed: A ruleset reported by someone who witnessed others who played the game, or who was taught the game by people of another culture.
  – Suggested: A ruleset for a historical game that was proposed by someone.
  – Incomplete: A ruleset which is known to only contain a partial set of rules. In most cases, this will be a ruleset with the rules that are compiled from DLP evidence.
  – Scholarly: A ruleset for a historical game proposed by an expert of that game.
  – Reconstructed: A ruleset reconstructed by the DLP.

**Table 11.** Structure of the RulesetTypes table.

Field	Type	Null	Key	Default	Extra	Foreign Key
Id	int(11)	NO	PRI	NULL	auto increment	
Name	varchar(45)	NO		NULL		

### Games and Rulesets Section

**Figure 5.**
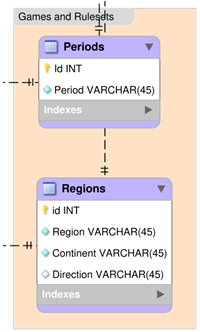
An Entity Relationship Diagram (ERD) for the Games and Rulesets group of the DLP database.

This section describes all the tables within the Games and Rulesets group of the DLP database, see Figure 5.

***Periods.*** Dimension table, describing possible periods for both games and rulesets (see Table 12). All values in this table are automatically updated by an external script.

- Id: Primary identifier (should be left blank).
- Period: Period name (Ancient if EvidenceRange < 500 CE, Medieval if 500 CE < EvidenceRange 1500 CE, Modern if EvidenceRange > 1500 CE).

**Table 12.** Structure of the Periods table.

Field	Type	Null	Key	Default	Extra	Foreign Key
Id	int(11)	NO	PRI	NULL	auto increment	
Period	varchar(45)	NO		NULL		

***Regions.*** Dimension table, describing possible Regions for both games and rulesets. All values in this table are automatically updated by an external script.

- Id: Primary identifier (should be left blank).
- Region: Name of the region. All Regions are represented as .kml files, which can be found in the evidence/worldRegions folder of the database (.zip) download file. The regions are based on the United Nations Statistical Division geoscheme, with the addition of the Northern Asia region, consisting of the portion of Russia which is east of the Ural Mountains.
- Continent: Continent of the region.
- Direction: Direction of the region (if applicable).

### Ludemes Section

**Figure 6.**
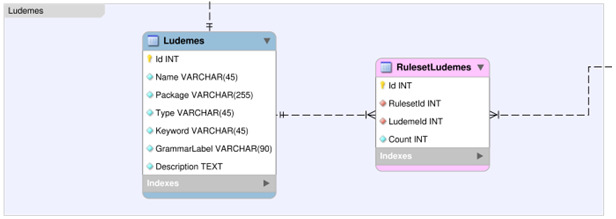
An Entity Relationship Diagram (ERD) for the Ludemes group of the DLP database.

This section describes all the tables within the Ludemes group of the DLP database, see Figure 6. See Table 13.

**Table 13.** Structure of the Regions table.

Field	Type	Null	Key	Default	Extra	Foreign Key
Id	int(11)	NO	PRI	NULL	auto increment	
Region	varchar(45)	NO		NULL		
Continent	varchar(45)	NO		NULL		
Direction	varchar(45)	YES		NULL		

***Ludemes.*** Fact table, describing each ludeme (see Table 14).

- Id: Primary identifier (should be left blank).
- Name: Name of this ludeme.
- Package: Package that this ludeme belongs to.
- Type: Types of ludeme (Ludeme/SuperLudeme/Constant/Predefined/Primitive).
- Keyword: Ludeme’s token as used in game descriptions.
- GrammarLabel: Grammar symbol corresponding to this ludeme.
- Description: Description of this ludeme.

**Table 14.** Structure of the Ludemes table.

Field	Type	Null	Key	Default	Extra	Foreign Key
Id	int(11)	NO	PRI	NULL	auto increment	
Name	varchar(45)	NO		NULL		
Package	varchar(255)	NO		NULL		
Type	varchar(45)	NO		NULL		
Keyword	varchar(45)	NO		NULL		
GrammarLabel	varchar(90)	NO		NULL		
Description	text	NO		NULL		

***RulesetLudemes.*** Junction table, describing pairings between rulesets and ludemes (see Table 15). This ties a ludeme to a ruleset.

- Id: Primary identifier (should be left blank).
- RulesetId: Primary identifier of the Ruleset table value associated with this pairing.
- LudemeId: Primary identifier of the Ludemes table value associated with this pairing.
- Count: The number of times this ludeme occurs in this ruleset’s description.

**Table 15.** Structure of the RulesetLudemes table.

Field	Type	Null	Key	Default	Extra	Foreign Key
Id	int(11)	NO	PRI	NULL	auto increment	
RulesetId	int(11)	NO	MUL	NULL		GameRulesets – Id
LudemeId	int(11)	NO	MUL	NULL		Ludemes – Id
Count	int(11)	NO	MUL	0		

### Evidence Section

**Figure 7.**
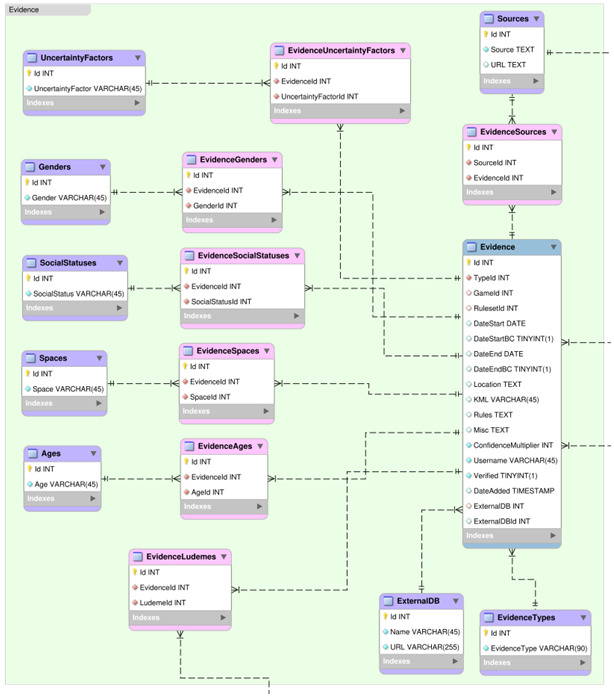
An Entity Relationship Diagram (ERD) for the Evidence group of the DLP database.

This section describes all the tables within the Evidence group of the DLP database, as shown in Figure 7.

***Evidence.*** Fact table, describing each piece of evidence, as shown in Table 16.

- Id: Primary identifier (should be left blank).
- TypeId: Primary identifier of the EvidenceType table value associated with this piece of Evidence. This assigns an Evidence Type to a particular piece of Evidence.
- GameId: Primary identifier of the Game table value associated with this piece of Evidence. This ties the Evidence to a particular Game.
- RulesetId: Primary identifier of the GameRulesets table value associated with this piece of Evidence. This ties the Evidence to a particular Ruleset, if possible.
- DateStart: This is the earliest possible date from which the information in this piece of Evidence comes, in the format YYYY-MM-DD. For analytical purposes, if left blank, it will be assigned the earliest date of any piece of Evidence for the relevant game.
- DateStartBC: True if the start date for this piece of evidence falls in the BC/BCE era of the Gregorian calendar.
- DateEnd: This is the latest possible date from which the information in this piece of Evidence comes, in the format YYYY-MM-DD. For analytical purposes, if left blank, it will be assigned the latest date of any piece of Evidence for the relevant game.
- DateEndBC: True if the end date for this piece of evidence falls in the BC/BCE era of the Gregorian calendar.
- Location: GPS coordinates for this piece of evidence in the form (6◦44’29.95”N, 44◦15’43.61”E). If multiple coordinates for a single piece of evidence, then these should be separated with a semi-colon. This place should indicate, as accurately as possible, the place from which the piece of evidence comes.
- KML: Name of the defined .kml region file associated with this piece of evidence. If multiple .kml files are associated with a single piece of evidence, then these should be separated with a semi-colon. All possible .kml region files can be found in the evidence/kml folder of the database (.zip) download file. A .kml should be used when a GPS coordinate is not sufficient for a piece of Evidence, either because the Evidence explicitly is about a region or because the exact location can only be estimated.
- Rules: These are the rules that are presented by this piece of evidence. Specific, individual rules as accurately as possible are to be placed here as they are demonstrated by the piece of Evidence, and not assumed based on other pieces of Evidence.
- Misc: Generally, the Misc field will describe the piece of evidence: either a short description of its content or its exact text, if available and concise. At least one reference should be cited, using AJA style. Anything relevant to identifying, assigning uncertainly, or qualifying statements about the piece of Evidence goes here.
- ConfidenceMultiplier: Confidence value between 0 and 100 for representing the reliability of this piece of evidence. This value is currently redundant, but may be used to store a multiplier for qualifying the confidence in the interpretation of the content of the piece of Evidence, if needed in future.
- Username: Ludii Forum username of the person who added this evidence into the DLP database.
- Verified: True if this piece of evidence has been verified by a member of the DLP team.
- DateAdded: The date that this piece of evidence was added to the DLP database (should be left blank).
- ExternalDB: Primary identifier of the ExternalDB table value associated with this piece of Evidence. This ties the Evidence to an external database.
- ExternalDBId: Identifying value for the corresponding entry in the external database referenced by the ExternalDB entry. This ties the Evidence to a specific entry within an external database.

**Table 16.** Structure of the Evidence table.

Field	Type	Null	Key	Default	Extra	Foreign Key
Id	int(11)	NO	PRI	NULL	auto increment	
TypeId	int(11)	NO	MUL	NULL		EvidenceTypes – Id
GameId	int(11)	NO	MUL	NULL		Games – Id
RulesetId	int(11)	YES	MUL	NULL		GameRulesets – Id
DateStart	date	YES		NULL		
DateStartBC	tinyint(1)	YES		0		
DateEnd	date	YES		NULL		
DateEndBC	tinyint(1)	YES		0		
Location	text	YES		NULL		
KML	varchar(45)	YES		NULL		
Rules	text	YES		NULL		
Misc	text	YES		NULL		
ConfidenceMultiplier	int(11)	NO		100		
Username	varchar(45)	NO		Walter.Crist		
Verified	tinyint(1)	NO		1		
DateAdded	timestamp	YES		CURRENT TIMESTAMP		
ExternalDB	int(11)	YES	MUL	NULL		ExternalDB – Id
ExternalDBId	int(11)	YES		NULL		

***EvidenceSources.*** Junction table, describing pairings between pieces of evidence and sources (see Table 17). This ties a Source (i.e., a bibliographic reference) to a piece of Evidence.

- Id: Primary identifier (should be left blank).
- SourceId: Primary identifier of the Sources table value associated with this pairing.
- EvidenceId: Primary identifier of the Evidence table value associated with this pairing.

**Table 17.** Structure of the EvidenceSources table.

Field	Type	Null	Key	Default	Extra	Foreign Key
Id	int(11)	NO	PRI	NULL	auto increment	
SourceId	int(11)	NO	MUL	NULL		Sources – Id
EvidenceId	int(11)	NO	MUL	NULL		Evidence – Id

***EvidenceAges.*** Junction table, describing pairings between pieces of evidence and ages (see Table 18). This ties an age (e.g. Child, Elder, Adolescent, Adult, etc.) to a piece of Evidence.

- Id: Primary identifier (should be left blank).
- EvidenceId: Primary identifier of the Evidence table value associated with this pairing.
- AgeId: Primary identifier of the Ages table value associated with this pairing.

**Table 18.** Structure of the EvidenceAges table.

Field	Type	Null	Key	Default	Extra	
Id	int(11)	NO	PRI	NULL	auto increment	
EvidenceId	int(11)	NO	MUL	NULL		Evidence – Id
AgeId	int(11)	NO	MUL	NULL		Ages – Id

***EvidenceSocialStatuses.*** Junction table, describing pairings between pieces of evidence and social statuses (see Table 19). This ties a social status (e.g. Non-Elite, Royalty, Clergy, Merchant, etc.) to a piece of Evidence.

- Id: Primary identifier (should be left blank).
- EvidenceId: Primary identifier of the Evidence table value associated with this pairing.
- SocialStatusId: Primary identifier of the SocialStatuses table value associated with this pairing.

**Table 19.** Structure of the EvidenceSocialStatuses table.

Field	Type	Null	Key	Default	Extra	
Id	int(11)	NO	PRI	NULL	auto increment	
EvidenceId	int(11)	NO	MUL	NULL		Evidence – Id
SocialStatusId	int(11)	NO	MUL	NULL		SocialStatuses – Id

***EvidenceSpaces.*** Junction table, describing pairings between pieces of evidence and spaces (see Table 20). This ties a space (e.g. Inside, Public, Ritual, Communal, etc.) to a piece of Evidence.

- Id: Primary identifier (should be left blank).
- EvidenceId: Primary identifier of the Evidence table value associated with this pairing.
- SpaceId: Primary identifier of the Spaces table value associated with this pairing.

**Table 20.** Structure of the EvidenceSpaces table.

Field	Type	Null	Key	Default	Extra	
Id	int(11)	NO	PRI	NULL	auto increment	
EvidenceId	int(11)	NO	MUL	NULL		Evidence – Id
SpaceId	int(11)	NO	MUL	NULL		Spaces – Id

***EvidenceLudemes.*** Junction table, describing pairings between pieces of evidence and ludemes (see Table 21). This ties a piece of evidence to a ludeme.

- Id: Primary identifier (should be left blank).
- EvidenceId: Primary identifier of the Evidence table value associated with this pairing.
- LudemeId: Primary identifier of the Ludemes table value associated with this pairing.

**Table 21.** Structure of the EvidenceLudemes table.

Field	Type	Null	Key	Default	Extra	
Id	int(11)	NO	PRI	NULL	auto increment	
EvidenceId	int(11)	NO	MUL	NULL		Evidence – Id
ludemeId	int(11)	NO	MUL	NULL		Ludemes – Id

***EvidenceUncertaintyFactors.*** Junction table, describing pairings between pieces of evidence and uncertainty factors (see Table 22). This ties an uncertainty factor (e.g. Incomplete, Graffiti, Ambiguous pattern, Uncertain date, etc.) to a piece of Evidence.

- Id: Primary identifier (should be left blank).
- EvidenceId: Primary identifier of the Evidence table value associated with this pairing.
- UncertaintyFactorId: Primary identifier of the UncertaintyFactors table value associated with this pairing.

**Table 22.** Structure of the EvidenceUncertaintyFactors table.

Field	Type	Null	Key	Default	Extra	
Id	int(11)	NO	PRI	NULL	auto increment	
EvidenceId	int(11)	NO	MUL	NULL		Evidence – Id
UncertaintyFactorId	int(11)	NO	MUL	NULL		UncertaintyFactors – Id

***EvidenceGenders***

Junction table, describing pairings between pieces of evidence and genders (see Table 23). This ties a genders (e.g. Female, Male, etc.) to a piece of Evidence.

- Id: Primary identifier (should be left blank).
- EvidenceId: Primary identifier of the Evidence table value associated with this pairing.
- GenderId: Primary identifier of the Genders table value associated with this pairing.

**Table 23.** Structure of the EvidenceGenders table.

Field	Type	Null	Key	Default	Extra	
Id	int(11)	NO	PRI	NULL	auto increment	
EvidenceId	int(11)	NO	MUL	NULL		Evidence – Id
GenderId	int(11)	NO	MUL	NULL		Genders – Id

***EvidenceTypes.*** Dimension table, describing possible types for pieces of evidence (see Table 24).

- Id: Primary identifier (should be left blank).
- EvidenceType: Name of the evidence type (e.g. Rules text, Artistic depiction, Artifact, Ethnogra- phy, etc.).

**Table 24.** Structure of the EvidenceTypes table.

Field	Type	Null	Key	Default	Extra	Foreign Key
Id	int(11)	NO	PRI	NULL	auto increment	
EvidenceType	varchar(90)	NO		NULL		

***ExternalDB.*** Dimension table, describing possible external databases for pieces of evidence (see Table 25).

- Id: Primary identifier (should be left blank).
- Name: Name of the external database.
- URL: URL for accessing the external database.

**Table 25.** Structure of the ExternalDB table.

Field	Type	Null	Key	Default	Extra	Foreign Key
Id	int(11)	NO	PRI	NULL	auto increment	
Name	varchar(45)	NO		NULL		
URL	varchar(255)	NO		NULL		

***Sources.*** Dimension table, describing possible sources for pieces of evidence (see Table 26).

- Id: Primary identifier (should be left blank).
- Source: Reference for the source (books, articles, works of art, manuscripts, etc.) in American Journal of Archaeology (AJA) style.
- URL: Website for assisting in accessing this source. When possible, this should be a link to the text or an image of the source itself. Otherwise, it must be a link to the item’s record at worldcat.org or another resource that will show the user where they can access this material in a library, museum, or other institution.

**Table 26.** Structure of the Sources table.

Field	Type	Null	Key	Default	Extra	Foreign Key
Id	int(11)	NO	PRI	NULL	auto increment	
Source	text	NO		NULL		
URL	text	YES		NULL		

***Ages.*** Dimension table, describing possible ages for pieces of evidence (see Table 27).

- Id: Primary identifier (should be left blank).
- Age: Name of the age. We use the categories Child, Adolescent, Adult, and Elder. These can be chosen as appropriate to the evidence, where provided.

**Table 27.** Structure of the Ages table.

Field	Type	Null	Key	Default	Extra	Foreign Key
Id	int(11)	NO	PRI	NULL	auto increment	
Age	varchar(45)	NO		NULL		

***SocialStatuses.*** Dimension table, describing possible social statuses for pieces of evidence (see Table 28).

- Id: Primary identifier (should be left blank).
- SocialStatus: The type of social status, which refers either to a socio-economic category, or some other kind of occupational grouping. These will, in many cases, overlap (e.g., Elite and Royalty).

**Table 28.** Structure of the SocialStatuses table.

Field	Type	Null	Key	Default	Extra	Foreign Key
Id	int(11)	NO	PRI	NULL	auto increment	
SocialStatus	varchar(45)	NO		NULL		

***Spaces.*** Dimension table, describing possible spaces for pieces of evidence (see Table 29).

- Id: Primary identifier (should be left blank).
- Space: These are spaces in which games were played, i.e., the kinds of buildings or outdoor settings in which gameplay actually occurred. These will overlap in many cases (e.g., Inside and Household).

**Table 29.** Structure of the Spaces table.

Field	Type	Null	Key	Default	Extra	Foreign Key
Id	int(11)	NO	PRI	NULL	auto increment	
Space	varchar(45)	NO		NULL		

***UncertaintyFactors.*** Dimension table, describing possible uncertainty factors for pieces of evidence, as shown in Table 30.

- Id: Primary identifier (should be left blank).
- UncertaintyFactor: An Uncertainty Factor is some aspect about the piece of evidence which indicates that its content is incomplete, that the attribution of the evidence to a particular game can be questioned, or that any piece of the information contained in that piece of evidence can be questioned. A piece of evidence may have none, or many, of these attached to it. Below are the factors currently used, though others may be added in the future:
  – Incomplete: Refers to an artefact or text which is not completely preserved.
  – Graffiti: Refers to information (usually a game board) which appears in the form of graffiti.
  – Ambiguous pattern: Refers to a piece of evidence for which the game referenced is unclear, or could apply to more than one game.
  – Uncertain date: Refers to a piece of evidence for which the original date of the object or text is unknown or in question.
  – Unknown provenience: Refers to a piece of evidence for which its place of origin is unknown or under question.
  – Written > 100 years after events: Refers to a piece of evidence depicting or describing an event more than one hundred years prior to the date of the text.
  – Written > 500 years after events: Refers to a piece of evidence depicting or describing an event more than five hundred years prior to the date of the text. When this uncertainty factor is chosen, Written > 100 years after events must also be chosen, because they are both true.
  – Written about a foreign culture: Refers to a piece of evidence that was written by someone discussing a culture other than their own.
  – Translated from another language: Refers to a non-English text.
  – Secondary Text: Refers to a source which describes another text or object, but which is not the actual text of that document. This may be, for example, a translation of a text reported in a book that is not primarily about that text.
  – Game not named: The name of the game is not explicitly included in the piece of evidence.
  – Unrepresentative depiction: Refers to a piece of evidence that depicts a game, but is not meant to be an exact depiction of it, for instance a. painting of chess with the wrong number of squares or pieces.
  – Insufficiently described rules: Refers to a description of a game that only contains a partial description of the rules.
  – Board not described: Refers to a description which omits a description of the board.
  – Rules not described: Refers to a description of a game which does not include the rules.
  – Rules learned second-hand: Refers to a piece of evidence where the person reporting the rules learned them from another person, who in turn learned them from someone in the culture to which the game belongs.
  – Suspected copy of earlier work: Refers to a piece of evidence that is suspected to be a copy of an earlier work, which has never been found.
  – Suspected forgery: Refers to an object of questionable authenticity.

**Table 30.** Structure of the UncertaintyFactors table.

Field	Type	Null	Key	Default	Extra	Foreign Key
Id	int(11)	NO	PRI	NULL	auto increment	
UncertaintyFactor	varchar(45)	NO		NULL		

***Genders.*** Dimension table, describing possible genders for pieces of evidence, as shown in Table 31.

- Id: Primary identifier (should be left blank).
- Gender: Male, or Female should be chosen when the game is either said to be played only by people of that gender, or only supplies information about people belonging to one of those genders. ”All” should be selected when the source states that there is no gender disparity in who plays the game. ”Other” stands in for other gender identities which may be identified as players of a particular game. This may be clarified and expanded as necessary.

**Table 31.** Structure of the Genders table.

Field	Type	Null	Key	Default	Extra	Foreign Key
Id	int(11)	NO	PRI	NULL	auto increment	
Gender	varchar(45)	NO		NULL		

##  Accessing the DLP Database

All data contained within, or referenced by, the DLP database is fully public and available to access.

### Complete Database

The complete DLP Database package can be downloaded as a single .zip file from: https://ludii.games/downloads/database.zip.

This includes the complete MySQL file (.sql) for describing the DLP database structure and data, which can be loaded into any MySQL administrator program (e.g. phpMyAdmin), as well as an evidence folder which contains other sub-folders of specific files referenced by the DLP database. This includes:

- kml: .kml region files referenced by the KML field in the Evidence table.
- pictures: various pictures that are associated with specific entries in the Evidence table (currently unorganised).
- worldRegions: .kml region files referenced by the Region field in the Regions table.
- lud: .lud game description files referenced by the Name field in the Games table.

### Individual Data Items

Specific entries within the DLP database are can be individually accessed using its unique identifier. The unique identifier for an entry is represented in the form DLP.T .N, where T is the name of the table within the DLP Database and N is the Id field value of the entry (e.g. DLP.Games.426 or DLP.Evidence.532). The field values associated with a unique identifier can be viewed using the link https://ludii.games/identifier?Id=DLP.T.N (e.g. https://ludii.games/identifier?Id=DLP.Games.426).

These unique identifiers should also be used when referencing a specific entry in the DLP database within an external document. When referencing a specific .lud game description file associated with a game, include both the reference for the game (DLP.Games.N ) and the version of the .lud file that was used (this can be found within the .lud file itself, and is the same as the Ludii App version number).

## DLP Server

**Figure 8.**
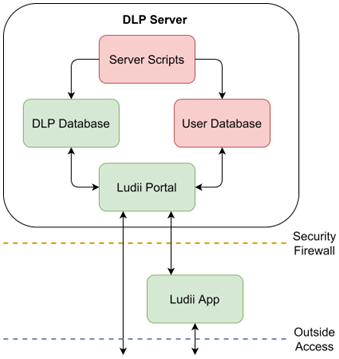
Overview of the DLP Server, what aspects it contains, and how these interact with each other. Boxes which are red contain private information, and cannot be directly accessed or downloaded in their entirety.

The DLP database that this document provides is stored within the DLP Server, which is housed at Maastricht University behind a secure firewall. However, the information within this Database is used and updated by many other processes. We therefore provide a short overview of the distinct processes and structures which are stored on the DLP Server and how they interact and communicate with each other, see Figure 8.

### DLP Database

The DLP Database contains all the information described in this document.

### User Database

The User Database contains personal information about the players who use Ludii. As the vast majority of the information in this database is private, it is not available to download. However, some information that is stored in this database can be viewed using either the Ludii Portal or Ludii App (tournament results, player statistics, usernames, etc.)

### Server Scripts

Several external scripts run periodically on the DLP Server to update information stored in the DLP and Ludii Databases. For the DLP Database, this includes the origin point and evidence ranges for each game. For the User Database, this includes logout timers, tournament results generation, Elo rating calculations, etc.

### Ludii Portal

The Ludii Portal consists of all websites and scripts under the ludii.games domain. This includes the game library, world map, forums, user account and database management, etc. The Ludii Portal communicates with the User Database for managing the Ludii Forum ( https://ludii.games/forums/), as well as providing several user account specific functions, such as tournament control panels or personalised recommendations. The Ludii Portal communicates with the the DLP database to access information about the games, rulesets and evidence it displays, as well as allowing select validated users to add new evidence to the DLP database.

### Ludii App

The Ludii App consists of the downloadable Ludii software in the form of a .jar executable. This includes functionality such as playing games, running AI-based analysis, participating in online tournaments, messaging other Ludii App users, etc. The Ludii App does not communicate with the DLP and Ludii Databases directly, but instead requests and sends information indirectly via the Ludii Portal, which then communicates with these databases. When engaging in network games or tournaments, the Ludii App communicates with the User Database to facilitate all remote connections and data transfer (verifying user identity, connecting to online games, sending moves and messages, etc.) The Ludii App also communicates with the DLP Database, sending information about the results of the experiments and analysis it conducts, and receiving metadata information about the games it contains (rulesets, description, origin, etc.).

## FAIR Compliance

The FAIR Principles are a set of guidelines established by a consortium of scientists and organisations to promote the preservation and effective reuse of scholarly data ^6, 8^. The four main FAIR Principles, and the ways in which the DLP Database and its constituent data adhere to these principles, are summarised below:

### Findable

Data should be assigned globally unique and persistent identifiers and be indexed in a searchable database.

Data in the five main data categories of the DLP Database – Games, Rulesets, Games and Rulesets, Ludemes and Evidence – each have a globally unique identifier that identifies their data category. For example, the identifier DLP.Games.7 uniquely identifies the game “20 Squares” as the seventh entry in the Games category of the DLP Database. The DLP Database is searchable in SQL format through the freely available database download1 and also manually searchable through the web-based Ludii Game Library ^2^ which provides user-friendly interface for navigating the games within the DLP Database and the associated evidence for them.

### Accessible

Data should be freely retrievable by their unique identifiers using standard communication protocols.

Individual data items within the DLP Database are freely accessible using standard internet protocols, by encoding relevant details about the item, including its data category and unique and index, as a URL in the format https://ludii.games/identifier?Id=DLP.T.N For example, accessing the DLP Server with the URL https://ludii.games/identifier?Id=DLP.Games.7 will retrieve the data for the game “20 Squares”.

### Interoperable

Data should use a formal, accessible, shared and applicable language for knowledge representation, and include qualified references to other data.

All data is stored as plain text to maximise compatibility and make it as widely reusable as possible.

We are able to utilise and import data from other sources. This is not an automated process, as other research projects gather different data and format it according to their own needs. Therefore, any data from an outside project must be reviewed and reformatted to fit our needs.

Recently, we incorporated data from the ERC-funded Locus Ludi project 3 to be imported into our evidence table. When the data was received, the data table was reformatted in R to eliminate irrelevant columns (data which they collect but which we do not) and entries (games which they are documenting but which we are not), add columns that are present in our table but absent from theirs (data they are not collecting; making sure these contain their default values), merging columns (when the data are presented in their table in multiple columns in theirs but in one in ours), reformat all data fields to be of the appropriate type, and reorder columns to match that of our data table.

A pertinent example involved the conversion of dates. In the Locus Ludi data, dates are recorded in two columns, dating start and dating end, as an integer, negative indicating a BCE date. These dates needed to be converted to YYYY-MM-DD format and the columns renamed to DateStart and DateEnd. New columns needed to be added, DateStartBC and DateEndBC, with a value of 1 for all BCE dates, and a value of 0 for all others. The columns were reordered: DateStart, DateStartBC, DateEnd, DateEndBC, within the table (following RulesetId and preceding Location) to maintain the order of our data table.

Once the data were converted to fit our table format as laid out in section 3 above, the new data were then converted to a CSV file, and imported into the Ludii database. Further editing within certain fields (such as the text in Content, which contains data merged from several Locus Ludii columns) was performed in PHPMyAdmin to more closely resemble the style of our descriptions.

Further work was necessary to link relevant tables (such as links between these new Evidence entries and Sources), which were performed as though they were newly generated entries.

The original identifiers of the Locus Ludii data (Name and URL) are recorded in the ExternalDB table. For each piece of evidence that was originally sourced from the Locus Ludii dataset, the database Id is given the ExternalDB field, and the unique identifier for that entry is placed in ExternalDBId.

Another simple type of data that is reused in the DLP Database from an outside source is the BGG identifier for each game. This is a unique identifier within the BoardGameGeek ^4^ online database for each game, in the format BGG.N (where N is the identifier number). BoardGameGeek is the world’s premier online database for board games and a wealth of non-scientific information including user ratings and game popularity, so it useful to correlate each game in the DLP Database with their corresponding entry (or entries) in the BGG database.

### Reusable

Data should be richly described with relevant attributes, released with a clear usage licence, and meet relevant community standards.

All data in the DLP Database is publicly available for reuse without restriction, with the exception of some categories of proprietary or potentially sensitive data that are kept private, e.g. rule set descriptions for proprietary games that we do not have permission to disseminate, and user data harvested from online play sessions that might have implications for GDPR compliance. Each data item in the DLP Database is stored in plain text format for maximum portability and each data field is labelled with a meaningful name to encourage its correct use.

## Data Availability

All data associated with this article can be accessed via the Accessing the DLP Database subsection. The complete DLP Database package can be downloaded as a single .zip file from: https://ludii.games/downloads/database.zip.

## Footnotes

^2^ https://ludii.games/library.php

^3^ https://locusludi.ch/the-project/

^4^ https://boardgamegeek.com
