# Supplementary material for: Data Note: The Digital Ludeme Project Database
Source: Open Res Eur. 2023 Sep 28;3:164. [Version 1] doi: 10.12688/openreseurope.16524.1 (PMC10973761; doi:10.12688/openreseurope.16524.1)
Supplement: Peer review history

# Reviewer report: Reviewer response for version 1

**DOI:** 10.21956/openreseurope.17838.r35370

**Published:** 2024-03-27

**Authors:** Jorge Nuno Silva, João Pedro Neto

**Affiliations:** 1 History and Philosophy of Science, University of Lisbon (Ringgold ID: 37809), Lisbon, Lisbon, Portugal; 2 University of California Berkeley, Berkeley, California, USA; 3 Computer Science, University of Lisbon (Ringgold ID: 37809), Lisbon, Lisbon, Portugal

The development of the Digital Ludeme Project (DLP) is, in the context of history of games, a major event, since 

 it provides a rational realm to present previously scattered and unclassified information.

 The inclusion of sophisticated computational methods and resources enriches the outputs of the project

 in a game-changing way.

 The new tools produced are useful in many an activity related to games: research, teaching, extension, etc.

 This article aims at explaining how DLP has been implemented, clarifying choices made in the process. Clearly, 

 it is successful at doing that.

 We list some observations, which we think should deserve the attention of the authors.

 Introduction section: it is stated that this document in an update, referring just one inclusion. Perhaps the writers should list other noticeable differences, to help readers already familiar with the previous version.

 Purpose section: are the secondary source assumptions mapped into the database? Or just information from primary sources?

 Game-Related section: it would not be impossible, by a major coincidence, that two different games could use the same ruleset. Is the data model able to represent that? The ERD seems to say yes, but perhaps it should be said, to underscore the differences between games and rulesets.

 Ruleset section: the sentence 'There are different types of rulesets.' feels out of place.

 If a game has several rulesets, is there one default ruleset? If yes, what are the criteria to choose it? (note: in page 7 the authors mention a «base ruleset»)

 The two sentences:

     This data group constitutes our mid-level description of games.

     This data group constitutes our low-level description of games.

 are perhaps placed in the wrong place?

 The ERD of page 7 should have more definition. It's very hard to read even by zooming in the pdf file (the tables are bigger in the following pages, but it would be nice to see them all together with proper detail)

 There is a ProprietaryGame and a PublicGame field. Is there any possibility that both are true, or both are false?

 Why the Credit field does not map into a Creator table? The database allows different names of the same person? The same question seems relevant for the Origin field. 

 And the EvidenceRange shouldn't it be two integers?

 Were the game categories predefined by humans (using standard names) or were they adjusted using a clustering process given their ludemes? It seems the former was adopted. Have you tried the latter? If so, a footnote would be interesting reading.

 What is the relevance of the Period table?

 On the Ludeme table, clarify what Package means. The model states it is a string, but what kind of information is inside that string?

 Clarify types of ludeme. Eg, what is a superludeme?

 What means «Grammar symbol corresponding to this ludeme»?

 URL https://ludii.games/downloads/database.zip does not work

 [outside the scope of this document] I tried using your services, and checking ludeme Gravity, https://ludii.games/ludemeDetails.php?Id=228, I was expecting the list to include Connect4 but it only has Gonnect. I think this is a typo in your data, or the ludeme means something else entirely.

Are sufficient details of methods and materials provided to allow replication by others?

Yes

Is the rationale for creating the dataset(s) clearly described?

Yes

Are the datasets clearly presented in a useable and accessible format?

Yes

Are the protocols appropriate and is the work technically sound?

Yes

Reviewer Expertise:

History of mathematics. History of boardgames.

We confirm that we have read this submission and believe that we have an appropriate level of expertise to confirm that it is of an acceptable scientific standard.

# Reviewer report: Reviewer response for version 1

**DOI:** 10.21956/openreseurope.17838.r35367

**Published:** 2024-03-27

**Authors:** Aris Politopoulos

**Affiliations:** 1 Leiden University, Leiden, The Netherlands

The article presents the complete outline the Digital Ludeme's Project database, as well as the rationale behind the type of data that were collected. 

 The rationale behind the creation of the database is clearly described under the section "purpose", although this particular section could be expanded a bit further (e.g. with one more paragraph) with some more citations and a few examples of the issues described. 

 The protocols are appropriate and the work is technologically and methodologically sound. 

 The article provides a full description of the database, as well as the choices that went behind certain elements of the database (e.g. the Ludemes as a baseline for describing and connecting rules).

 Datasets are clearly presented and described with consistent examples to illustrate the functionalities of the database.

Are sufficient details of methods and materials provided to allow replication by others?

Yes

Is the rationale for creating the dataset(s) clearly described?

Yes

Are the datasets clearly presented in a useable and accessible format?

Yes

Are the protocols appropriate and is the work technically sound?

Yes

Reviewer Expertise:

Archaeology of play, ancient board games, archaeogaming, digital archaeology

I confirm that I have read this submission and believe that I have an appropriate level of expertise to confirm that it is of an acceptable scientific standard.
